# Association of Medical Students' Reports of Interactions with the Pharmaceutical and Medical Device Industries and Medical School Policies and Characteristics: A Cross-Sectional Study

**DOI:** 10.1371/journal.pmed.1001743

**Published:** 2014-10-14

**Authors:** James S. Yeh, Kirsten E. Austad, Jessica M. Franklin, Susan Chimonas, Eric G. Campbell, Jerry Avorn, Aaron S. Kesselheim

**Affiliations:** 1 Program On Regulation, Therapeutics, And Law (PORTAL), Division of Pharmacoepidemiology and Pharmacoeconomics, Department of Medicine, Brigham and Women's Hospital and Harvard Medical School, Boston, Massachusetts, United States of America; 2 Center on Medicine as a Profession, Columbia University, New York, New York, United States of America; 3 Mongan Institute for Health Policy, Massachusetts General Hospital and Harvard Medical School, Boston, Massachusetts, United States of America; York University, Canada

## Abstract

**Background:**

Professional societies use metrics to evaluate medical schools' policies regarding interactions of students and faculty with the pharmaceutical and medical device industries. We compared these metrics and determined which US medical schools' industry interaction policies were associated with student behaviors.

**Methods and Findings:**

Using survey responses from a national sample of 1,610 US medical students, we compared their reported industry interactions with their schools' American Medical Student Association (AMSA) PharmFree Scorecard and average Institute on Medicine as a Profession (IMAP) Conflicts of Interest Policy Database score. We used hierarchical logistic regression models to determine the association between policies and students' gift acceptance, interactions with marketing representatives, and perceived adequacy of faculty–industry separation. We adjusted for year in training, medical school size, and level of US National Institutes of Health (NIH) funding. We used LASSO regression models to identify specific policies associated with the outcomes. We found that IMAP and AMSA scores had similar median values (1.75 [interquartile range 1.50–2.00] versus 1.77 [1.50–2.18], adjusted to compare scores on the same scale). Scores on AMSA and IMAP shared policy dimensions were not closely correlated (gift policies, *r = *0.28, 95% CI 0.11–0.44; marketing representative access policies, *r = *0.51, 95% CI 0.36–0.63). Students from schools with the most stringent industry interaction policies were less likely to report receiving gifts (AMSA score, odds ratio [OR]: 0.37, 95% CI 0.19–0.72; IMAP score, OR 0.45, 95% CI 0.19–1.04) and less likely to interact with marketing representatives (AMSA score, OR 0.33, 95% CI 0.15–0.69; IMAP score, OR 0.37, 95% CI 0.14–0.95) than students from schools with the lowest ranked policy scores. The association became nonsignificant when fully adjusted for NIH funding level, whereas adjusting for year of education, size of school, and publicly versus privately funded school did not alter the association. Policies limiting gifts, meals, and speaking bureaus were associated with students reporting having not received gifts and having not interacted with marketing representatives. Policy dimensions reflecting the regulation of industry involvement in educational activities (e.g., continuing medical education, travel compensation, and scholarships) were associated with perceived separation between faculty and industry. The study is limited by potential for recall bias and the cross-sectional nature of the survey, as school curricula and industry interaction policies may have changed since the time of the survey administration and study analysis.

**Conclusions:**

As medical schools review policies regulating medical students' industry interactions, limitations on receipt of gifts and meals and participation of faculty in speaking bureaus should be emphasized, and policy makers should pay greater attention to less research-intensive institutions.

*Please see later in the article for the Editors' Summary*

## Abstract 2

Aaron Kesselheim and colleagues compared US medical students' survey responses regarding pharmaceutical company interactions with the schools' AMSA PharmFree scorecard and Institute on Medicine as a Profession's (IMAP) scores.

*Please see later in the article for the Editors' Summary*

## Editors' Summary

**Background:**

Making and selling prescription drugs and medical devices is big business. To promote their products, pharmaceutical and medical device companies build relationships with physicians by providing information on new drugs, by organizing educational meetings and sponsored events, and by giving gifts. Financial relationships begin early in physicians' careers, with companies providing textbooks and other gifts to first-year medical students. In medical school settings, manufacturers may help to inform trainees and physicians about developments in health care, but they also create the potential for harm to patients and health care systems. These interactions may, for example, reduce trainees' and trained physicians' skepticism about potentially misleading promotional claims and may encourage physicians to prescribe new medications, which are often more expensive than similar unbranded (generic) drugs and more likely to be recalled for safety reasons than older drugs. To address these and other concerns about the potential career-long effects of interactions between medical trainees and industry, many teaching hospitals and medical schools have introduced policies to limit such interactions. The development of these policies has been supported by expert professional groups and medical societies, some of which have created scales to evaluate the strength of the implemented industry interaction policies.

**Why Was This Study Done?:**

The impact of policies designed to limit interactions between students and industry on student behavior is unclear, and it is not known which aspects of the policies are most predictive of student behavior. This information is needed to ensure that the policies are working and to identify ways to improve them. Here, the researchers investigate which medical school characteristics and which aspects of industry interaction policies are most predictive of students' reported behaviors and beliefs by comparing information collected in a national survey of US medical students with the strength of their schools' industry interaction policies measured on two scales—the American Medical Student Association (AMSA) PharmFree Scorecard and the Institute on Medicine as a Profession (IMAP) Conflicts of Interest Policy Database.

**What Did the Researchers Do and Find?:**

The researchers compared information about reported gift acceptance, interactions with marketing representatives, and the perceived adequacy of faculty–industry separation collected from 1,610 medical students at 121 US medical schools with AMSA and IMAP scores for the schools evaluated a year earlier. Students at schools with the highest ranked interaction policies based on the AMSA score were 63% less likely to accept gifts as students at the lowest ranked schools. Students at the highest ranked schools based on the IMAP score were about half as likely to accept gifts as students at the lowest ranked schools, although this finding was not statistically significant (it could be a chance finding). Similarly, students at the highest ranked schools were 70% less likely to interact with sales representatives as students at the lowest ranked schools. These associations became statistically nonsignificant after controlling for the amount of research funding each school received from the US National Institutes of Health (NIH). Policies limiting gifts, meals, and being a part of speaking bureaus (where companies pay speakers to present information about the drugs for dinners and other events) were associated with students' reports of receiving no gifts and of non-interaction with sales representatives. Finally, policies regulating industry involvement in educational activities were associated with the perceived separation between faculty and industry, which was regarded as adequate by most of the students at schools with such policies.

**What Do These Findings Mean?:**

These findings suggest that policies designed to limit industry interactions with medical students need to address multiple aspects of these interactions to achieve changes in the behavior and attitudes of trainees, but that policies limiting gifts, meals, and speaking bureaus may be particularly important. These findings also suggest that the level of NIH funding plays an important role in students' self-reported behaviors and their perceptions of industry, possibly because institutions with greater NIH funding have the resources needed to implement effective policies. The accuracy of these findings may be limited by recall bias (students may have reported their experiences inaccurately), and by the possibility that industry interaction policies may have changed in the year that elapsed between policy grading and the student survey. Nevertheless, these findings suggest that limitations on gifts should be emphasized when academic medical centers refine their policies on interactions between medical students and industry and that particular attention should be paid to the design and implementation of policies that regulate industry interactions in institutions with lower levels of NIH funding.

**Additional Information:**

Please access these websites via the online version of this summary at http://dx.doi.org/10.1371/journal.pmed.1001743.

- The UK General Medical Council provides guidance on financial and commercial arrangements and conflicts of interest as part of its good medical practice document, which describes what is required of all registered doctors in the UK
- Information about the American Medical Student Association (AMSA) Just Medicine campaign (formerly the PharmFree campaign) and about the AMSA Scorecard is available
- Information about the Institute on Medicine as a Profession (IMAP) and about its Conflicts of Interest Policy Database is also available
- “Understanding and Responding to Pharmaceutical Promotion: A Practical Guide” is a manual prepared by Health Action International and the World Health Organization that medical schools can use to train students how to recognize and respond to pharmaceutical promotion
- The US Institute of Medicine's report “Conflict of Interest in Medical Research, Education, and Practice” recommends steps to identify, limit, and manage conflicts of interest
- The ALOSA Foundation provides evidence-based, non-industry-funded education about treating common conditions and using prescription drugs

## Introduction

Interactions between health care professionals and the prescription drug and medical device industries are common, especially in academic medical centers [1]. Such relationships can be controversial in the context of medical education and physician training [2]–[6]. Several studies have documented the interactions between drug and device companies and trainees at all levels, from providing textbooks and other gifts to first-year medical students to funding continuing medical education (CME) programs for practitioners [7]–[10]. Critics have charged that such interactions can impart biased information and may contribute to a “hidden curriculum” that reduces trainees' skepticism about potentially misleading promotional claims [11]–[16]. Studies have shown that this biased information can increase non-evidence-based prescribing and increase the cost of patient care [17]–[19].

To address these concerns, in the past decade numerous teaching hospitals and medical schools in the US have sought to isolate trainees from industry through policies limiting the activities of marketing representatives on campus [20]–[24]. Development of such policies has been supported by expert professional groups and medical societies such as the Institute of Medicine, the Association of American Medical Colleges, the American Board of Internal Medicine Foundation, the American Medical Student Association (AMSA), and the Institute on Medicine as a Profession (IMAP). Among these, AMSA and IMAP have also created scales—respectively, the AMSA PharmFree Scorecard and the IMAP Conflicts of Interest Policy Database—to evaluate the strength of institutions' policies around these issues [25]–[27].

In a recent survey of first-year and fourth-year US medical students, Austad et al. showed that trainees express substantial enthusiasm for limiting the involvement of industry marketing in medical education, although trainee acceptance of commercially sponsored gifts and meals has continued [25]. The survey also found that the level of US National Institutes of Health (NIH) research funding at a given medical school was a strong predictor of student behavior, with students at schools receiving NIH research support above the median institutional funding level less likely to report receiving gifts from industry than students at non-research-intensive schools. However, the impact of other institutional factors on trainees' behavior remains unclear.

We used data from Austad et al.'s survey to further investigate the relationship between trainees' interactions with industry representatives and key characteristics of their medical schools, particularly the strength of policies regarding interactions between institutions and industry. Our goal was to determine which medical school characteristics and which conflict of interest policy dimensions were most predictive of students' reported behaviors.

## Methods

### Ethics Statement

This study was approved by the Institutional Review Board at Brigham and Women's Hospital and Harvard Law School.

### Study Population and Survey Administration

Survey design and administration were described in detail by Austad et al. [25]. In brief, a nationwide random sample of US medical students was identified from the American Medical Association Masterfile (*n = *3,495; 14 first-year students each from 120 US allopathic medical schools [one school had missing data for first-year students] and 15 fourth-year students each from 121 US allopathic medical schools; 231 medical students were excluded from the analysis because of inaccurate mailing addresses). Here, we focus on three issues previously reported to be of importance [28] reflecting students' marketing interactions with industry and students' learning environments: (1) students' reported receipt of gifts and meals, (2) their discussions with pharmaceutical representatives about drug products, and (3) perceptions of the adequacy of separation between medical school faculty members and the pharmaceutical industry. We considered responses to the following questions.

1. “Which of the following have you received in the last six months from drug, device, or other medically related companies?” Five options were listed: “food or beverage in the workplace,” “free drug samples,” “a meal outside of campus or hospital,” “pens, notepads, T-shirts,” and “any other gift or financial support.” A positive response to any of these was considered as receipt of a gift.
2. “Which of the following interactions have you experienced in the past six months?” A “yes” response to “talked with a pharmaceutical representative about a company's products” was coded as having interacted with a marketing representative.
3. “In your experience, how much do you agree or disagree with the following statements regarding your institution?” One statement offered was “I believe that there is adequate separation between the Faculty of Medicine at my university and the pharmaceutical industry.” Possible answers were “strongly agree,” “agree,” “disagree,” and “strongly disagree.” We dichotomized responses between respondents who answered “strongly agree” and “agree” versus those who answered “disagree” or “strongly disagree.”

The survey provided a US$2 honorarium [29] and received responses from 1,610 first-year and fourth-year medical students (representing a 49.3% response rate).

### Medical School–Industry Interactions Ranking Data

The policy dimensions evaluated by the AMSA PharmFree Scorecard and the IMAP Conflicts of Interest Policy Database fall into three categories of regulation: (1) individual–industry interactions, (2) institutional–industry interactions, and (3) industry involvement in educational activities. In 2010, the AMSA PharmFree Scorecard evaluated 11 dimensions of medical schools' industry interaction policies and assigned a value from one to three, with one being the lowest score possible (least restrictive) and three being the highest (most restrictive) [25]. The 11 dimensions were as follows: gifts, consulting, speaking bureaus, disclosure, marketing representative access, samples, purchasing/formulary committee participation, travel compensation, on-site educational events, scholarships, and medical school curriculum. To determine an overall “AMSA score,” we calculated the mean industry interaction policy score for each school using the 2010 data from AMSA across these 11 dimensions.

Next, as part of its 2010 PharmFree Scorecard, AMSA assigned a letter grade to each school on an A/B/C/D/F scale, with A reflecting the tightest controls on industry interaction. For each school, AMSA calculated this letter grade by taking the total cumulative score of the policy dimension scores of marketing representative access, samples, purchasing/formulary committee participation, and the top three policy dimension scores in each of the following two categories of regulation: individual–industry interactions (dimensions were gifts, consulting, speaking bureaus, and disclosure) and industry involvement in educational activities (dimensions were travel compensation, on-site educational events, scholarships, and medical school curriculum). The remaining fourth dimension with the lowest score in each of these categories was not calculated by AMSA as part of the cumulative score. The total cumulative score was then divided by the total possible score and multiplied by 100 to give a percentage. For percentages ≥85%, ≥70% and <85%, ≥60% and <70%, ≥40% and <60%, and <40%, AMSA assigned letter grades of A, B, C, D, and F, respectively. For each school, we converted the letter grade to a numerical value, with A–F corresponding to 4–0, to arrive at an “AMSA grade.”

Also in 2010, IMAP evaluated 12 policy dimensions on a 0–3 scale: meals, gifts, consulting, honoraria, speaking bureaus, marketing representative access, samples, purchasing/formulary committee participation, travel compensation, continuing medical education, scholarships, and ghostwriting [26]. We calculated an overall “IMAP score” as the average score on these 12 dimensions.

### Statistical Analysis

We calculated summary statistics for each policy score and each scored dimension, including medians and interquartile ranges (IQRs). We also calculated the linear correlation in overall AMSA score and IMAP score, as well as scores assigned to policy dimensions that were shared between the two criteria, including policies on industry gifts, marketing representative access, drug samples, and faculty consulting [30]. We considered a correlation coefficient *r = *0 to 0.25 to signify no association, *r = *0.26 to 0.5 to indicate a fair association, *r = *0.51 to 0.75 to indicate a moderate association, and *r*>0.75 to indicate a strong association [30].

We fit several hierarchical logistic regression models to estimate the association between these policy scores and students' self-reported behavior or perceptions. All models included a binary indicator of the behavior or perception as the dependent variable, a random intercept for medical school, and a fixed linear effect for the policy score. For each of the three survey questions and each industry interaction policy record (AMSA grade, AMSA score, and IMAP score), we estimated the association of the school's policy score with trainees' reported outcomes with increasing levels of adjustment, including (1) no adjustment, (2) adjustment for student year in training (first versus fourth), (3) adjustment for year in training and quartiles of medical school size, based on student enrollment, and (4) adjustment for year in training, medical school size, and a binary indicator of NIH funding, split at the median value (US$94.2 million) in 2010.

To compare the AMSA grade, AMSA score, and IMAP score despite their different value ranges, we transformed the estimated odds ratios (ORs) from the logistic regression models to represent the magnitude of difference in trainees' responses between institutions with the most restrictive policies (e.g., highest possible scores) to those with the least restrictive policies (e.g., lowest possible scores), even if no schools actually achieved these scores. The transformed AMSA score, IMAP score, and AMSA grade required the raising of the estimated OR to the second power, third power, and fourth power, respectively. We also estimated the corresponding probabilities of responses to industry interaction questions for students from schools with the most and least restrictive policies. For a direct comparison of the calculated mean values of the AMSA grade, AMSA score, and IMAP score, the AMSA grade and AMSA score were recalibrated to the same 0–3 scale used in the IMAP score.

Finally, we estimated two exploratory logistic regression models to identify the specific policy dimensions from each score that were most strongly associated with student behaviors for the three outcomes of interest: receiving gifts, interaction with marketing representatives, and perceived adequacy of faculty and industry separation. The first model used all IMAP dimensions as independent variables in a model predicting student behavior, and the second model used all AMSA dimensions as the independent variables. We used the LASSO (least absolute shrinkage and selection operator) approach, a penalized likelihood method for model estimation that performs simultaneous variable selection and coefficient estimation to produce a parsimonious list of predictors [31],[32]. We used the glmnet package in R (version 3.0.0, R Project for Statistical Computing), and implemented LASSO with cross-validation in order to choose an appropriate penalty parameter.

## Results

### Characteristics of Medical Schools

Of the 121 US allopathic medical schools analyzed in this study, 39% (*n = *47) were classified as private institutions. At the time of the survey, a “typical” US medical school enrolled a median number of 639 students, received US$94 million in NIH research funds, and had 890 faculty members (Table 1). The AMSA score ranked 63% (*n = *76) of the schools as having the most restrictive category of industry interaction policies (score >2 to 3), while the IMAP score ranked 67% (*n = *81) of school policies in the intermediate restrictive level (score >1 to 2).

**Table 1:** Medical school characteristics (*n = *121).

Characteristic	Result
**Median (IQR) number of enrolled students**	639 (478, 761)
**Median (IQR) annual NIH research funding (million US dollars)**	94.2 (28.1, 196.9)
**Median (IQR) size of medical school faculty**	890 (477, 1,405)
**IMAP score, proportion of school distribution**	
Least restrictive policies	13% (*n = *16)
Intermediate restrictive policies	63% (*n = *76)
Most restrictive policies	24% (*n = *29)
**AMSA score, proportion of school distribution**	
Least restrictive policies	6% (*n = *7)
Intermediate restrictive policies	27% (*n = *33)
Most restrictive policies	67% (*n = *81)

Least restrictive policies correspond to IMAP and AMSA scores of 0 to 1; intermediate restrictive policies are scores >1 to 2; most restrictive policies correspond to scores >2 to 3.

### Correlation of Responses to Outcomes of Interest

Students who reported interacting with a marketing representative were more than twice as likely to have reported receiving a gift (relative risk [RR] 2.60, 95% CI 2.39–2.95, *p*<0.0001) than students who did not interact with a marketing representative. There was no significant association between students receiving gifts and students' perception of adequacy of faculty separation from the industry (RR 0.92, 95% CI 0.77–1.09) or between students' interaction with a marketing representative and the students' perception of adequacy of faculty separation from the industry (RR 1.00, 95% CI 0.78–1.29).

### Characteristics of AMSA and IMAP Ranking Systems

The dimensions that comprised the 2010 AMSA and IMAP ranking systems are reported in Table 2, along with summary statistics for the overall scores. The AMSA and IMAP ranking systems consisted of three general categories of regulation: (1) individual–industry interactions, (2) institutional–industry interactions, and (3) industry involvement in educational activities. There were eight overlapping policy dimensions and three and four non-overlapping dimensions, respectively, in the AMSA and IMAP ranking systems. The IMAP score had a median value (1.75, IQR 1.50–2.00) similar to that of the AMSA score (1.77, IQR 1.50–2.18).

**Table 2:** Comparison of industry interaction policy dimensions in the AMSA and IMAP ranking systems.

Scoring Dimension	Dimension Definition	Median (IQR) AMSA Value	Median (IQR) IMAP Value	Correlation Coefficient (95% CI)
**Individual–industry interactions**				
Meals	Acceptance of meals paid for by the industry	n/a	2 (2, 3)	—
Gifts	Acceptance of gifts of any value^a^	3 (1.5, 3)	2 (2, 3)	0.28 (0.11, 0.44)
Consulting	Consulting relationships, excluding scientific research and speaking	1.5 (1.5, 3)	1 (1, 2)	0.59 (0.46, 0.70)
Honoraria	Financial compensation given for services provided that traditionally do not require compensation	n/a	1 (0, 2)	—
Speaking bureaus	Financial compensation for speaking on behalf of companies at conferences and educational events	1.5 (0, 1.5)	1 (1, 2)	0.52 (0.37, 0.63)
Disclosure	Disclosure of financial relationships with the industry	1.5 (0, 1.5)	n/a	—
**Institutional–industry interactions**				
Marketing representative access	Interaction with sales representatives	1.5 (1.5, 1.5)	2 (2, 2)	0.51 (0.36, 0.63)
Samples	Receipt of drug samples or vouchers for patient use	1.5 (0, 1.5)	1 (1, 3)	0.51 (0.37, 0.63)
Purchasing/formulary committee participation	Limitations on individuals with industry financial ties serving on purchasing or formulary committees	3 (1.5, 3)	0 (0, 2)	0.48 (0.31, 0.60)
**Industry involvement in educational activities**				
Travel compensation	Acceptance of industry financial support to attend meetings and educational events	3 (1.5, 3)	2 (1, 3)	0.45 (0.30, 0.58)
On-site educational events	Industry-sponsored events held on campus	1.5 (1.5, 1.5)	n/a	—
CME	Industry sponsorship of CME events	n/a	1 (1, 1)	—
Scholarships	Industry earmarking or sponsoring training of a specific individual	1.5 (0, 1.5)	3 (3, 3)	0.47 (0.32, 0.60)
Medical school curriculum	Medical student training on institutional conflict of interest policies	1.5, (0, 3)	n/a	n/a
Ghostwriting	Written work published under the name of health care personnel that was written in part or in full by pharmaceutical industry staff or paid writers	n/a	0 (0,3)	—
**Overall score**		**1.77 (1.5, 2.18)**	**1.75 (1.5, 2)**	**0.69 (0.59, 0.78)**

a The AMSA PharmFree Scorecard includes meals in this category.

n/a, not applicable.

The AMSA grade, determined by the nine best-scoring AMSA dimensions out of 11 possible dimensions, had the highest median value (2.25, IQR 1.50–2.25), indicating that it was the least stringent score of the three evaluated.

The linear correlations among the policy dimensions shared by AMSA and IMAP are also reported in Table 2; overall, they demonstrated only fair to moderate correlations, even though they assessed similar policies. The scoring of gift policies (*r = *0.28, 95% CI 0.11–0.44), purchasing and formulary committee policies (*r = *0.48, 95% CI 0.31–0.60), travel compensation policies (*r = *0.45, 95% CI 0.30–0.58), and scholarship policies (*r = *0.47, 95% CI 0.32–0.60) had fair correlations between AMSA and IMAP. We found stronger correlations for consulting policies (*r = *0.59, 95% CI 0.46–0.70), speaking bureau policies (*r = *0.52, 95% CI 0.37–0.63), marketing representative access policies (*r = *0.51, 95% CI 0.36–0.63), and sample policies (*r = *0.51, 95% CI 0.37–0.63), although the 95% CIs largely overlapped between those classified as fair versus moderate according to correlation coefficients.

The pairwise correlation between the three different ranking systems showed a moderate to strong relationship among the overall scores. Unsurprisingly, the AMSA grade and AMSA score demonstrated the strongest correlation (*r = *0.88, 95% CI 0.84–0.92). This was followed by IMAP score and AMSA score (*r = *0.69, 95% CI 0.59–0.78), and IMAP score and AMSA grade (*r = *0.51, 95% CI 0.37–0.63).

### Association of Scores with Student Responses

**Figure 1:**
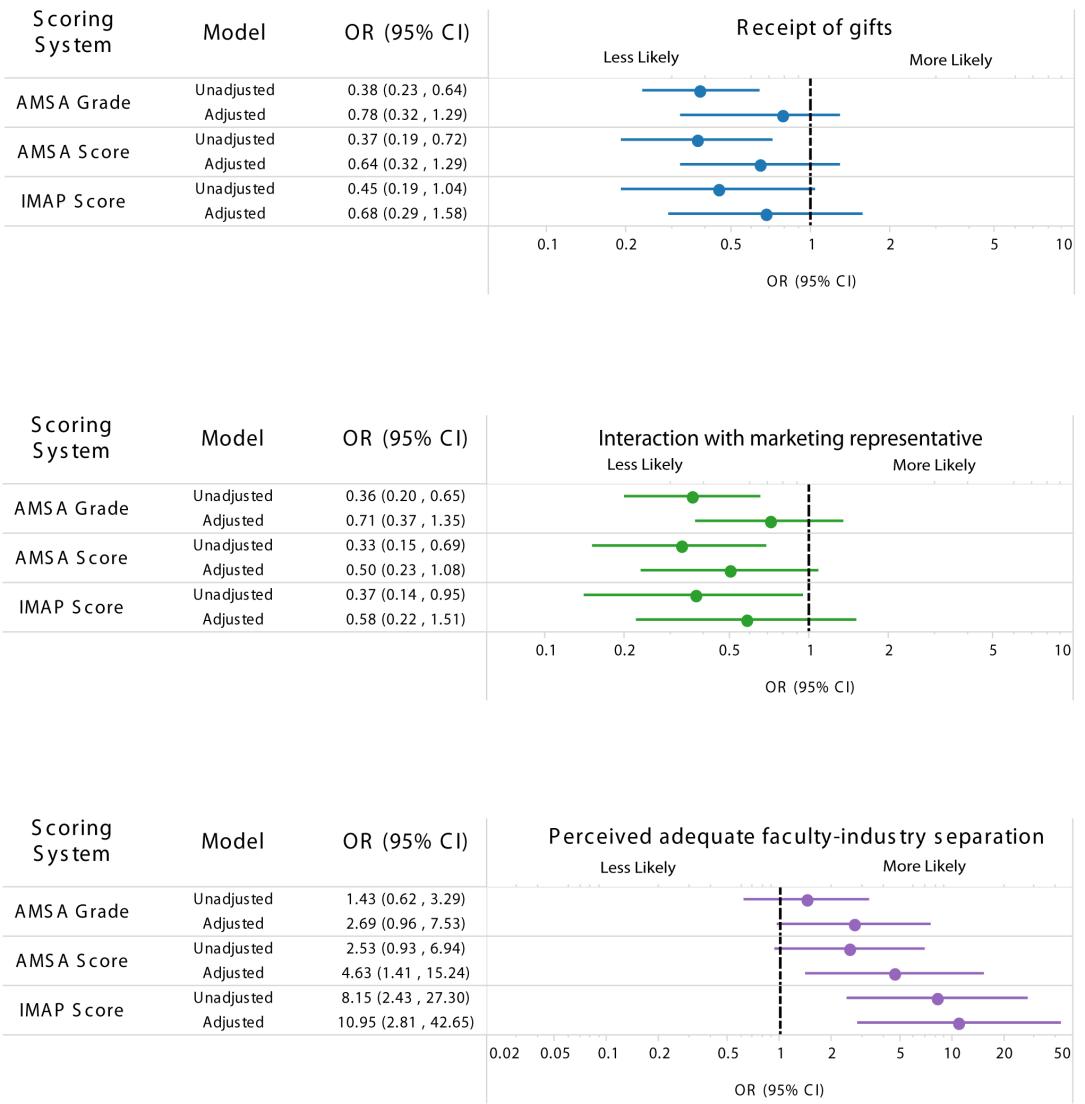
Association between strength of medical school industry interaction policies and survey responses. Outcome comparing schools with the most restrictive policies and schools with the least restrictive policies for receipt of gifts (top), interaction with marketing representatives (middle), and perceived adequacy of faculty–industry separation (bottom). For example, in the unadjusted AMSA grade scoring system, students from schools with the most restrictive policies had about 60% less odds of receiving gifts compared to those from a school with the least restrictive policies. The fully adjusted model included the year in training, size of the medical student population, and a dichotomous measure of NIH funding. NIH funding is a measure of the amount of government-funded basic science research occurring at the medical school. High NIH funding was defined as above the median value (US$94.2 million) for all medical schools in 2010 (compared to schools below the median value).

The associations of student responses with institutional policies are presented in Figure 1. In the unadjusted model, students were less likely to report receiving a gift from a marketing representative in schools with a higher AMSA grade (unadjusted OR 0.38, 95% CI 0.23–0.64) or an AMSA score indicating tighter policies (unadjusted OR 0.37, 95% CI 0.19–0.72). The IMAP score was also related to this outcome, but was not statistically significant (unadjusted OR 0.45, 95% CI 0.19–1.04). When we adjusted for year in training and medical school size in partially adjusted models, the relationship did not change substantially. However, adding NIH funding level to the fully adjusted model attenuated the relationship between all three policy scores and the outcome of receiving an industry gift (AMSA grade, fully adjusted OR [aOR] 0.78, 95% CI 0.32–1.29; AMSA score, aOR 0.64, 95% CI 0.32–1.29; IMAP score, aOR 0.68, 95% CI 0.29–1.58). The unadjusted ORs correspond to estimates of 56%–70% for the likelihood of receiving any industry-sponsored gifts for students from schools with the least possible restrictive policies compared to 37%–46% for students from schools with the most possible restrictive policies. This effect suggests that a school's NIH funding level is an important confounder of the relationship between policies, whether measured by AMSA or IMAP, and student behavior.

The outcome of meeting with marketing representatives showed a similar pattern. Students were 50%–70% times less likely to interact with marketing representatives in schools with higher scores on the AMSA and IMAP ranking systems (AMSA grade, unadjusted OR 0.36, 95% CI 0.20–0.65; AMSA score, unadjusted OR 0.33, 95% CI 0.15–0.69; IMAP score, unadjusted OR 0.37, 95% CI 0.14–0.95). The partially adjusted models did not alter this relationship. Again, including NIH funding level in the fully adjusted model substantially diminished the association with school policy, such that the relationships were no longer statistically significant (AMSA grade, aOR 0.71, 95% CI 0.37–1.35; AMSA score, aOR 0.50, 95% CI 0.23–1.08; IMAP score, aOR 0.58, 95% CI 0.22–1.51). Unadjusted ORs correspond to estimates of 47%–59% and 23%–32% for the likelihood of discussing industry products with marketing representatives for students at schools with the most and least restrictive policies, respectively.

Students' perceptions of the adequacy of faculty–industry separation at their institutions showed an opposite pattern for the AMSA systems. In the unadjusted and partially adjusted models, there was a nonsignificant relationship between the AMSA grade and AMSA score and students' perceptions about faculty–industry separation at their medical schools. The ORs correspond to an estimated 80%–91% and 93%–97% likelihood of believing there is adequate separation between the faculty and industry at schools with the most and least restrictive policies, respectively. However, in the fully adjusted model including NIH funding, the AMSA score was significantly associated with the strength of the policy (aOR 4.63, 95% CI 1.41–15.24).

By contrast, the IMAP score was significantly associated with students' reports of adequate separation between faculty and industry in both the unadjusted model (OR 8.15, 95% CI 2.43–27.30) and the fully adjusted model (aOR 10.95, 95% CI 2.81–42.65).

We also explored other ways of modeling NIH funding (e.g., in quartiles) and other institutional characteristics (e.g., private versus public school and the size of medical school faculties) and consistently found that NIH funding level was a significant confounder of the association between school policies and self-reported student behavior and attitudes (Figure S1).

### Predictors of Student Responses

The policy dimensions selected by the LASSO regression models as most predictive of each student response outcome are shown in Table 3. As this analysis is exploratory and because accurate estimates of model uncertainty are not straightforward from LASSO, we do not report coefficient values and confidence intervals; instead, we focus on which dimensions were selected for model inclusion based on their explanatory value in the model. Many of the policies governing individual–industry relationships, such as those limiting gifts, meals, and speaking bureaus, were associated with students' reports of receiving no gifts and not interacting with marketing representatives. Policy dimensions associated with the regulation of industry involvement in educational activities (e.g., CME, travel compensation, and scholarships) were not associated with answers to questions relating to receiving gifts or marketing representative interactions, but were associated with perceived separation between faculty and industry. Regulation of gifts was the only policy area consistently associated with students' reports of not receiving gifts and not interacting with marketing representatives, and with increased students' perception of faculty's adequacy of separation from the industry.

**Table 3:** Policy dimensions selected by LASSO as predictors to student responses.

Student Response to Survey Question	AMSA Dimensions Regarding Policies	IMAP Dimensions Regarding Policies
Receipt of gift	Gifts ↓Speaking bureaus ↓Purchasing/formulary committee ↓	Meals ↓Purchasing/formulary committee ↓
Interaction with a representative	Gifts ↓Consulting ↓Speaking bureaus ↓	Speaking bureaus ↓
Perceived adequate separation between faculty and industry	Purchasing/formulary committee ↑Travel compensation ↑	Gifts ↑Samples ↑CME ↑Consulting ↓Honoraria ↓Travel compensation ↑Scholarships ↑

The direction of the arrow indicates whether the presence of a school policy is associated with student agreement with the survey question (arrow pointing up) or disagreement with the survey question (arrow pointing down). For example, the AMSA gift policy dimension was associated with reduced reported receipt of gifts.

## Discussion

In the past decade, many US medical schools have instituted policies regulating student and faculty interactions with the pharmaceutical and device industries, but few studies have analyzed the effects of these policies on trainees' attitudes and behaviors. We found that students at schools with more rigorous industry interaction policies, as measured by both AMSA and IMAP rating methods, were significantly more likely to report they had not received gifts in the past 6 mo, had not interacted with industry marketing representatives in the past 6 mo, and perceived adequate separation of faculty and industry at their schools. We also found that NIH research funding was a strong predictor and important confounding variable with regard to the outcomes of receiving gifts and reporting interactions with marketing representatives. However, level of NIH funding was not a confounder for the outcome of students' perception of adequate separation between faculty and the pharmaceutical industry, which was associated with both AMSA and IMAP scores.

A number of possible explanations exist for the association of NIH funding level with students' self-reported behaviors and perceptions concerning industry. First, institutions with greater NIH funding likely have more experience with industry interaction policies. The NIH has long paid close attention to conflicts of interest among its funded investigators [33]. Therefore, when pressure—from sources such as students or the media—arose to reduce industry interactions such as gifts and meals, schools with a history of NIH funding and other government contracts had more experience in instituting and implementing such rules. Another possibility is that the enactment and implementation of industry interaction policies requires substantial resources, including dedicated compliance officers, that are more likely to be found in resource-rich schools. By contrast, schools with lower levels of NIH funding may be more dependent on industry financial support for their educational programs and overall budget. Thus, institutions with lower NIH funding levels may have disincentives to developing strong policies insulating students and faculty members from industry.

Student responses concerning the adequacy of separation between the school faculty and industry were likely influenced most by the very existence of industry interaction policies, as compared to the other two questions that reflected the actual execution of the policies. If an academic medical center has a strong industry relations policy but enforces it poorly or continues to permit interactions off campus or at satellite hospitals and clinics, then students at that institution would likely respond to our survey questions by perceiving adequate separation of their faculty while still reporting receipt of gifts and other interactions with industry. According to the AMSA scoring system, nearly three-quarters (74%) of schools in 2010 had oversight policies in place to ensure compliance, while 26% did not. In addition, the majority of schools (68%) reported mechanisms to sanction noncompliance with the regulations. As industry interaction policies continue to evolve, the policies should be designed to cover all affiliated student rotations and be meaningfully enforced.

Given that the AMSA and IMAP scoring systems covered overlapping components of medical schools' industry interaction policies, we expected that they would behave similarly in our analyses, but they differed in some cases. It is possible that each scoring system measures policies through different evaluative schema. Generally, we found that the mean AMSA score was more precise than the AMSA grade, which is consistent with the fact that the grade does not evaluate the entire component score. The AMSA PharmFree Scorecard program revised its scoring procedures this year; subsequent national surveys should assess whether the new version better accounts for implementation of school policies and better predicts students' receipt of gifts and interactions with industry [34].

We found that restricting receipt of gifts was the single interaction policy most often related to the outcomes we assessed. Policies banning gifts were associated not only with reduced reports of receipt of industry gifts by students, but also with fewer interactions with pharmaceutical marketing representatives overall and greater perception of adequate separation between the faculty and industry. These results suggest that as US academic medical centers look to create or reform regulations on industry interactions for medical students, limiting receipt of gifts should be a central feature of the policies. Medical trainees who receive even small-value gifts from marketing representatives have been found to have more favorable attitudes towards pharmaceutical products and marketing representatives and tend to believe they are immune to the biases that can arise from such interactions [12],[13],[28],[35],[36]. A recent study by Mintzes and colleagues pointed out that such interactions can lead to important negative clinical outcomes [37]. Institutional policies regulating industry-sponsored educational activities, such as stringent limits on industry-sponsored CME, travel, and scholarships, seem to exert the most influence over trainees' perceptions of the adequacy of separation between their schools' faculties and the pharmaceutical industry. These types of educational activities are particularly high-profile and may draw trainees' attention to industry's involvement in education [38].

Our study is limited by recall and other social biases inherent to survey studies. In addition, our study was cross-sectional in nature, and thus does not take into account recent changes that institutions might have made to their industry interaction policies. Student behavior is likely shaped by informal and formal school curricula, which are both likely related to the strength of industry interaction policies at a particular school. Since the survey was conducted in 2011 and the analysis was performed using the 2010 AMSA and IMAP databases, some curricula may have been altered by the time of the survey administration. However, changes in institutional culture are often slow, which may delay the adoption and implementation of conflict of interest policies [27]. Assessment of the scoring systems studied assumes that the dimensions measured by these policies are validated and adequately capture the numerous factors that predict trainee–industry interactions. In spite of this, the policy dimensions measured by the AMSA and IMAP ranking systems reflect attempts at regulating the most common industry marketing practices [39],[40]. Our study population was composed of a national sample of trainees, thus minimizing the threat to the external validity of our findings and making the findings more generalizable. The response rate was within the range of response rates achieved in studies of medical professionals [41],[42]. Numerous sensitivity checks performed on the respondent population showed no evidence of sampling bias [25].

Industry interaction policies at academic medical centers are intended to insulate trainees from the biases that such interactions can create [18],[19]. Our study demonstrates that multiple policy dimensions are associated with trainee-reported behaviors and attitudes, though certain policies, such as gift-giving, appear most fundamental. Furthermore, the level of NIH funding, which may serve as a proxy for familiarity with conflict of interest regulations, strongly influences the association between student reports of industry interactions and policies regarding student–industry interactions. As academic medical centers limit industry access, industry marketing representatives may focus efforts on less research-intensive institutions that permit greater access to the next generation of physicians [43]–[46]. Leaders in medical education will need to continue to assess the nature and effectiveness of institutional policies.

## Supporting Information

Figure S1 **Sensitivity analysis of association of strength of school interaction policies and student survey responses.** Outcome comparing schools with the most restrictive policies and schools with the least restrictive policies for receipt of gifts (top), interaction with marketing representatives (middle), and perceived adequacy of faculty–industry separation (bottom). Model 1 adjusts for year in training, size of medical student population, and a quartile split of 2010 NIH funding level. Model 2 adjusts for year in training, size of medical student population, quartile split of 2010 NIH funding level, quartile split of size of medical school faculty, and status as a private institution.
(TIF)

Table S1 **Uncalibrated AMSA score.**
(XLSX)

## Abbreviations

AMSA: American Medical Student Association
aOR: adjusted odds ratio
CME: continuing medical education
IMAP: Institute on Medicine as a Profession
IQR: interquartile range
NIH: US National Institutes of Health
OR: odds ratio
RR: relative risk
