# Supplementary figures and images for: Association of Medical Students' Reports of Interactions with the Pharmaceutical and Medical Device Industries and Medical School Policies and Characteristics: A Cross-Sectional Study
Source: PLoS Med. 2014 Oct 14;11(10):e1001743. doi: 10.1371/journal.pmed.1001743 (PMC4196737; doi:10.1371/journal.pmed.1001743)

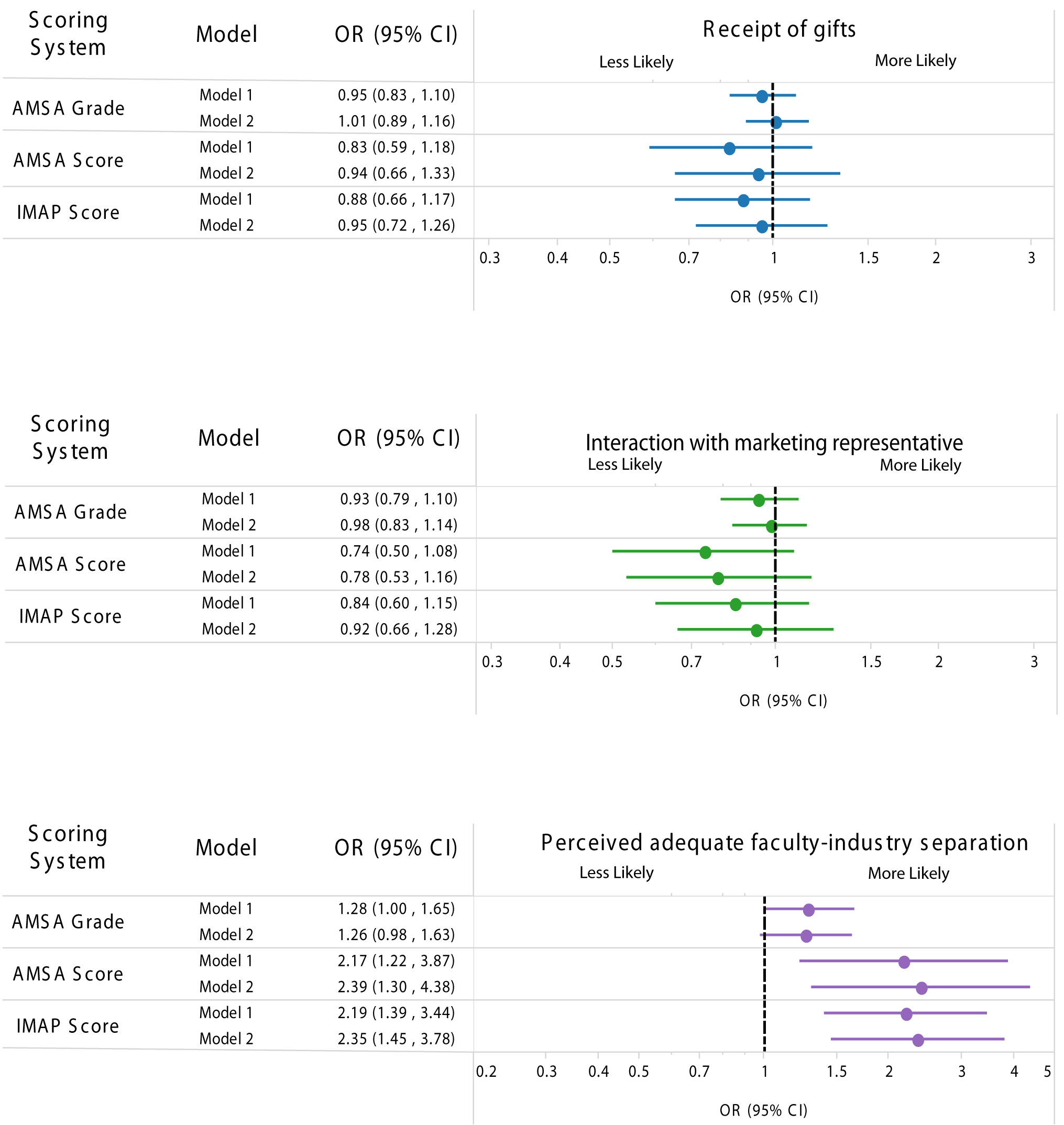

Supplement: Figure S1 — Sensitivity analysis of association of strength of school interaction policies and student survey responses. Outcome comparing schools with the most restrictive policies and schools with the least restrictive policies for receipt of gifts (top), interaction with marketing representatives (middle), and perceived adequacy of faculty–industry separation (bottom). Model 1 adjusts for year in training, size of medical student population, and a quartile split of 2010 NIH funding level. Model 2 adjusts for year in training, size of medical student population, quartile split of 2010 NIH funding level, quartile split of size of medical school faculty, and status as a private institution. (TIF) [file pmed.1001743.s001.tif]
